# Case Report: Clinical impact of *BRCA1* and *BRIP1* vs. *BRCA1* and *BRCA2* germline double heterozygosity in ovarian cancer: a comparative case study

**DOI:** 10.3389/fonc.2025.1614373

**Published:** 2025-07-24

**Authors:** Limei Zheng, Qianyuan Zhu, Fenglan Zhang, Hao Qiu, Lan Qin, Jianhua Yang, Ming Qi

**Affiliations:** ^1^ Department of Obstetrics and Gynecology in Sir Run Shaw Hospital, School of Medicine, Zhejiang University, Key Laboratory of Reproductive Dysfunction Management of Zhejiang Province, Hangzhou, China; ^2^ Polytechnic Institute of Zhejiang University, Hangzhou, Zhejiang, China; ^3^ Center for Clinical Genetics and Genomics, DIAN Diagnostics, Hangzhou, Zhejiang, China; ^4^ Assisted Reproduction Unit, Department of Obstetrics and Gynecology, Department of Laboratory Medicine, Sir Run Shaw Hospital, Zhejiang University School of Medicine, Key Laboratory of Reproductive Dysfunction Management of Zhejiang Province, Hangzhou, China; ^5^ DIAN Diagnostics, Hangzhou, Zhejiang, China; ^6^ Center for Precision Medicine, Zhejiang-California International NanoSystems Institute, Hangzhou, China; ^7^ Department of Pathology and Laboratory of Medicine, University of Rochester Medical Centre, Rochester, NY, United States

**Keywords:** BRCA1, BRCA2, BRIP1, germline double heterozygosity, ovarian cancer, case report

## Abstract

Ovarian cancer (OC) is a highly heterogeneous malignancy influenced by germline genetic factors, with *BRCA1/2* mutations being well-established risk factors. Germline double heterozygosity (GDH), particularly involving rare combinations, remains poorly understood. This study presents the first report of *BRCA1/BRIP1* GDH in a case of Chinese OC patients and compares their clinical characteristics and treatment responses to a patient with *BRCA1/BRCA2* GDH. The *BRCA1/BRIP1* GDH patient is a 46-year-old female diagnosed with advanced ovarian adenocarcinoma at clinical stage FIGO IVB, exhibited severe chemotherapy-induced toxicity and postoperative complications, including chylous leakage. In contrast, the *BRCA1/BRCA2* GDH patient is a 44-year-old female with high-grade serous ovarian cancer at clinical stage FIGO IIIC, tolerated chemotherapy well. Both patients experienced clinical benefit from Olaparib maintenance therapy. Genetic testing confirmed pathogenic variants in both cases, revealing distinct clinical trajectories influenced by different GDH profiles. Our findings suggest that different GDH combinations may influence chemotherapy tolerance and therapeutic effectiveness in OC. *BRCA1/BRIP1* GDH patients may require personalized dose adjustments to mitigate toxicity and optimize efficacy. This study underscores the clinical significance of GDH heterogeneity and the importance of comprehensive genetic testing for guiding individualized treatment strategies. Future research should focus on expanding sample sizes and conducting in-depth functional analyses to further clarify the clinical implications of different GDH types, ultimately refining treatment approaches for GDH-associated OC.

## 1 Introduction

Ovarian cancer (OC) is a heterogeneous group of malignancies originating in the ovaries, fallopian tubes, or peritoneum. It is the eighth leading cause of cancer-related mortality in women worldwide, with over 310,000 new cases and approximately 200,000 deaths reported in 2020 (1). Germline genetic factors play a significant role in the onset of breast cancer (BC) and OC. Carriers of pathogenic *BRCA1* and *BRCA2* variants are known to have an elevated risk of developing OC. The lifetime risk of OC in the general population is approximately 1.3% (2). Notably, the Homologous Recombination Repair (HRR) gene family is essential for genomic stability and DNA damage repair. According to the National Comprehensive Cancer Network (NCCN) (https://www.nccn.org/guidelines/category_2), different genetic variants are associated with varying risks of epithelial ovarian cancer (EOC), with *BRCA1* variants conferring an absolute risk of 39–58%, *BRCA2* variants 13–29%, and *BRIP1* variants 5–15%.

*BRCA1* and *BRCA2* are critical regulators of multiple cellular processes, including transcriptional regulation, cell cycle control, and the DNA damage response. They play a pivotal role in DNA repair via homologous recombination. *BRIP1*, which interacts with *BRCA1*, is essential for maintaining genomic integrity and functions as a tumor suppressor (3). Variants in *BRCA1* and *BRCA2* disrupt the HRR pathway, compromising the ability of cancer cells to repair platinum-induced DNA damage and thereby increasing their sensitivity to platinum-based chemotherapy. Previous studies, based on both preclinical models and clinical data, have demonstrated that carriers of *BRCA* or *BRIP1* gene variants exhibit significantly enhanced sensitivity to platinum-based chemotherapy, with notable improvements in overall survival (OS) (4, 5).

Most individuals carrying cancer susceptibility gene variants, such as *BRCA1*, *BRCA2*, and *ATM*, are heterozygous for a single pathogenic variant. Germline double heterozygosity (GDH) in OC and BC is rare. The advancement of sequencing technologies, particularly Next-Generation Sequencing (NGS), has facilitated the widespread adoption of multi-gene panel testing, leading to an increased number of reported GDH cases in recent years. GDH cases most commonly involve *BRCA1* and *BRCA2*, with BC being the predominant associated malignancy (6–8). In terms of racial distribution, most GDH reports involve individuals of European descent (7, 9–11), while among Asian populations, South Korea has the highest reported incidence (8, 12, 13). GDH cases in China have been reported infrequently (14–16).

In this study, we describe two Chinese OC patients carrying different GDH variants (*BRCA1/BRIP1* and *BRCA1/BRCA2*) and evaluate their chemotherapy tolerance, postoperative recovery, and responses to targeted therapy. The results show that the patient with the *BRCA1/BRIP1* variants experienced greater chemotherapy toxicity and developed postoperative chylous leakage. In contrast, the patient with the *BRCA1/BRCA2* mutation exhibited better chemotherapy tolerance. Notably, this study represents the first report of a *BRCA1/BRIP1* GDH variant in a Chinese OC patient.

## 2 Manuscript formatting

### 2.1 The result of patient 1

In December 2021, a 46-year-old woman presented to a local hospital with abdominal distension. She had no significant past medical history. Cytological analysis of ascites confirmed adenocarcinoma, and PET-CT revealed a right adnexal mass with metastases involving the mesentery, left supraclavicular region, mediastinum, bilateral internal mammary regions, multiple subcapsular hepatic lymph nodes, and possible sacral bone involvement. The clinical stage was FIGO IVB. Due to limited medical resources during the COVID-19 pandemic, the patient was not a candidate for primary surgery and instead received individualized palliative chemotherapy at the local hospital (paclitaxel 260 mg, cisplatin 60 mg, and bevacizumab 900 mg) for five cycles. Although no imaging assessment was performed after these cycles. According to the patient’s self-report, there was significant symptomatic improvement, suggesting a partial response. Chemotherapy was discontinued due to severe gastrointestinal side effects, and the patient subsequently switched to oral traditional Chinese medicine.

In March 2023, the patient was referred to our hospital for further management. Repeat PET-CT demonstrated persistent disease with a similar extent of metastasis. She then received three cycles of neoadjuvant chemotherapy (paclitaxel and carboplatin). Follow-up imaging revealed more than a 30% reduction in tumor size, consistent with a partial response according to RECIST criteria. On May 17, 2023, the patient underwent open abdominal cytoreductive surgery for OC, including total hysterectomy, bilateral salpingo-oophorectomy, omentectomy, pelvic lymphadenectomy, presacral lymphadenectomy, and para-aortic lymphadenectomy. Residual lymph nodes (<1 cm) remained in the left supraclavicular fossa, right internal mammary region, and hepatic hilum, achieving R1 resection. Postoperatively, the patient received three additional cycles of paclitaxel, carboplatin, and bevacizumab, with the final cycle administered on August 2, 2023. Multiple dose adjustments were necessary due to significant hematologic toxicities ranging from grade 1 to 4. The postoperative period was complicated by chylous leakage, requiring specialized management. The patient is currently undergoing maintenance therapy with Olaparib. Over 17 months of outpatient follow-up, no new lesions have been detected, and the residual lymph nodes have remained stable. The disease remains stable.

**Figure 1:**
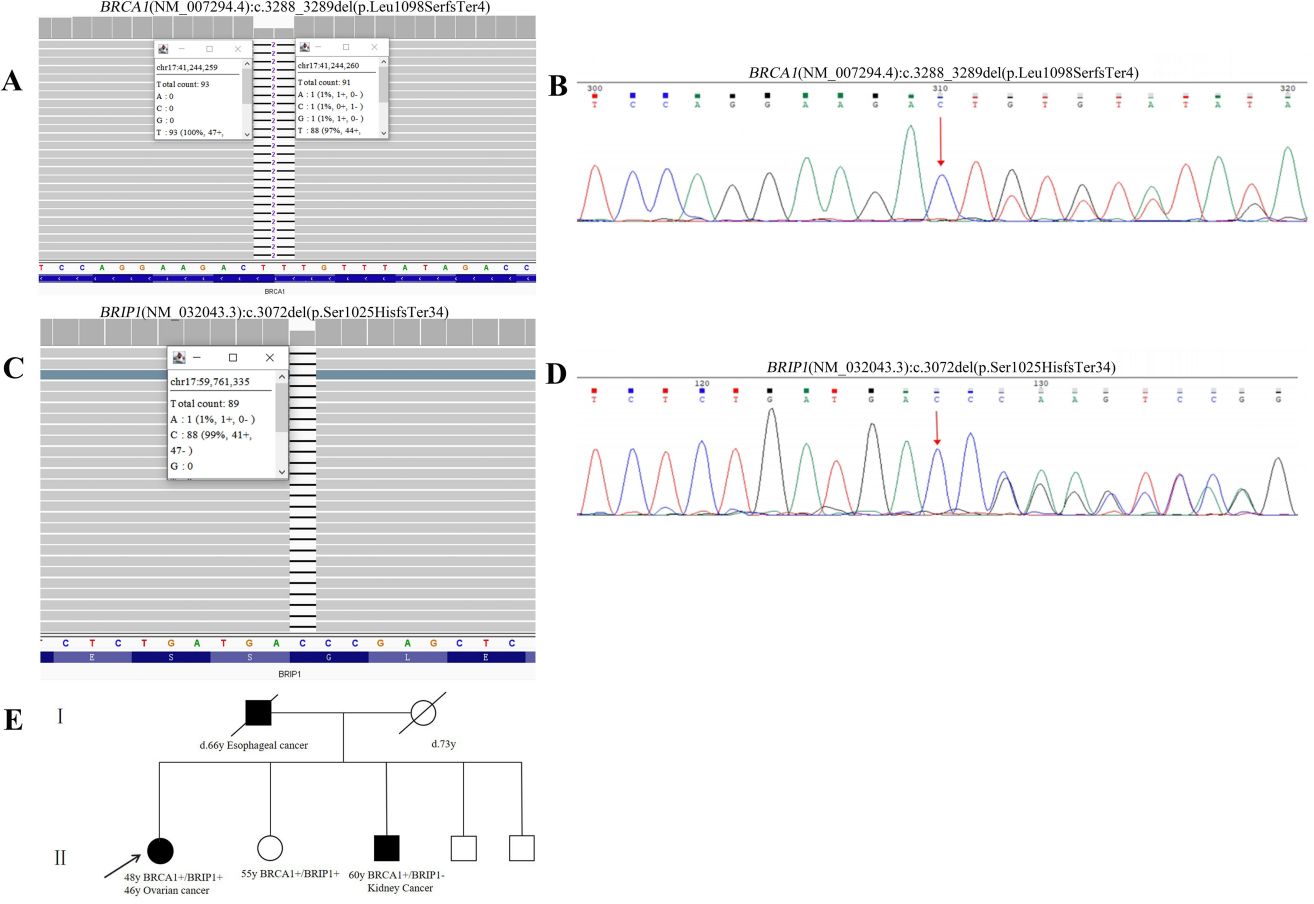
Pedigree and molecular test results of Patient 1. **(A)** WES data of *BRCA1* (NM_007294.4):c.3288_3289del (p.Leu1098SerfsTer4) pathogenic variant. **(B)** Sanger sequencing result of the *BRCA1* pathogenic variant. **(C)** WES data of *BRIP1* (NM_032043.3):c.3072del (p.Ser1025HisfsTer34) likely pathogenic variant. **(D)** Sanger sequencing result of the *BRIP1* likely pathogenic variant. Red arrows indicate the identified variants. **(E)** Pedigree of individual 1. Black indicates individuals with cancer. Cancer type and age at diagnosis are reported. The age for genetic testing and age of death are also reported. Family members carrying the pathogenic/likely pathogenic variant are marked with *BRCA1*+, *BRIP1*+.

**Figure 2:**
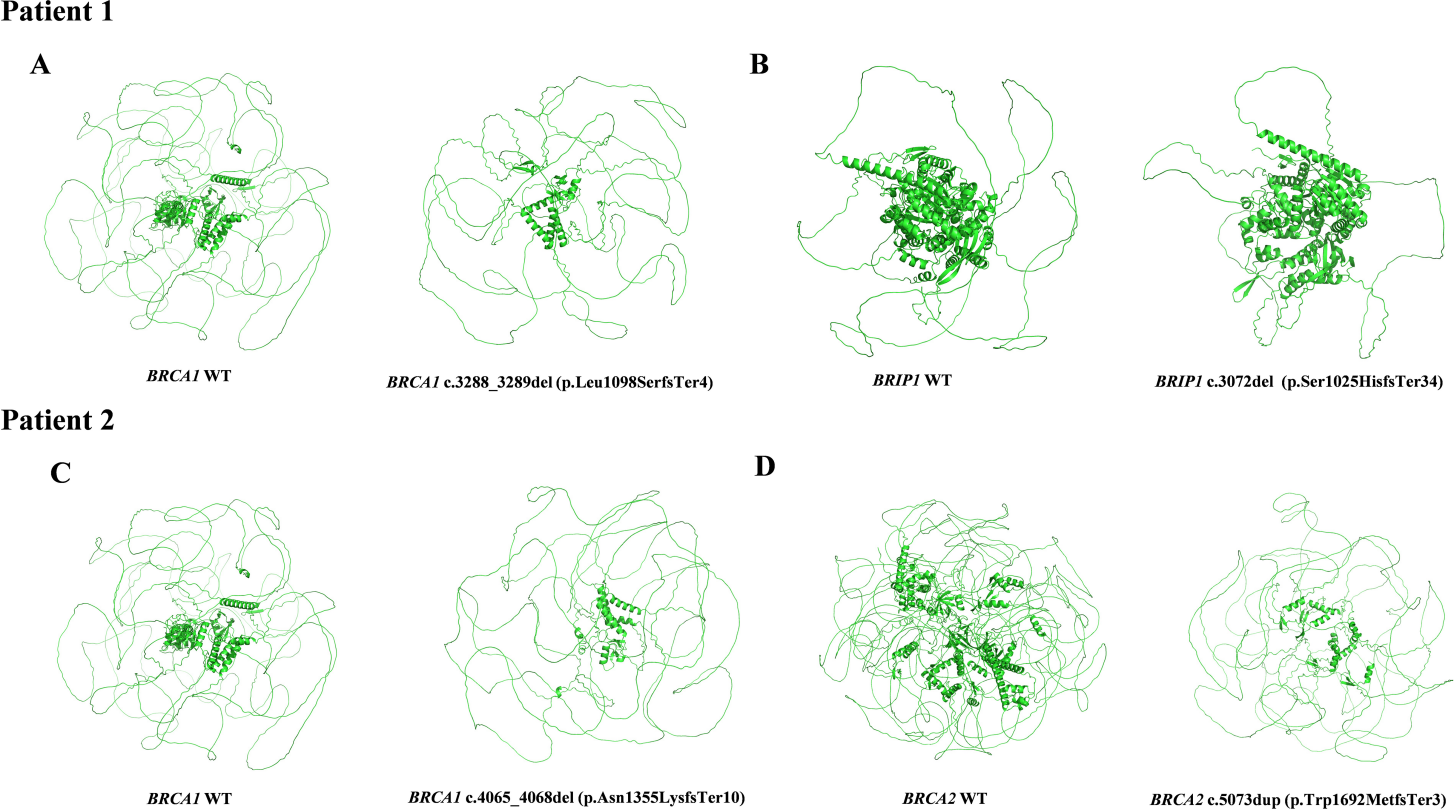
3D structures of wild-type and variant proteins identified in Patient 1 and Patient 2. **(A)** *BRCA1* WT vs. *BRCA1* (NM_007294.4):c.3288_3289del (p.Leu1098SerfsTer4) 3D Structures in Patient 1. **(B)** *BRIP1* WT vs. *BRIP1* (NM_032043.3):c.3072del (p.Ser1025HisfsTer34) 3D Structures in Patient 1. **(C)** *BRCA1* WT vs. *BRCA1* (NM_007294.4):c.4065_4068del (p.Asn1355LysfsTer10) 3D Structures in Patient 2. **(D)** *BRCA2* WT vs. *BRCA2* (NM_000059.3):c.5073dup (p.Trp1692MetfsTer3) 3D Structures in Patient 2.

The Whole exome sequencing (WES) testing identified GDH variants in the patient, including (NM_007294.4) c.3288_3289del (p.Leu1098SerfsTer4) in exon 17 of *BRCA1* and (NM_032043.3) c.3072del (p.Ser1025HisfsTer34) in exon 17 of *BRIP1* (**Figures 1A-D**). According to the guidelines of the American College of Medical Genetics and Genomics (ACMG) (17), the *BRCA1* variant was classified as pathogenic (PVS1+PS4+PM2_P), while the *BRIP1* variant was classified as likely pathogenic (PVS1+ PM2_P). To further illustrate the potential structural impact of these variants, three-dimensional protein structure models were generated and are shown in **Figures 2A, B**.

The proband’s father passed away from esophageal cancer at the age of 66, while her mother died of cerebral infarction at the age of 73. Among her siblings, one brother has kidney cancer, and her unaffected sister shares the same GDH as the proband. The brother with kidney cancer carries a single *BRCA1* (NM_007294.4) c.3288_3289del (p.Leu1098SerfsTer4) variant. Based on familial genetic testing, the two germline variants were determined to be of paternal and maternal origin. **Figure 1E** shows the genealogic tree of this family.

### 2.2 The result of patient 2

**Figure 3:**
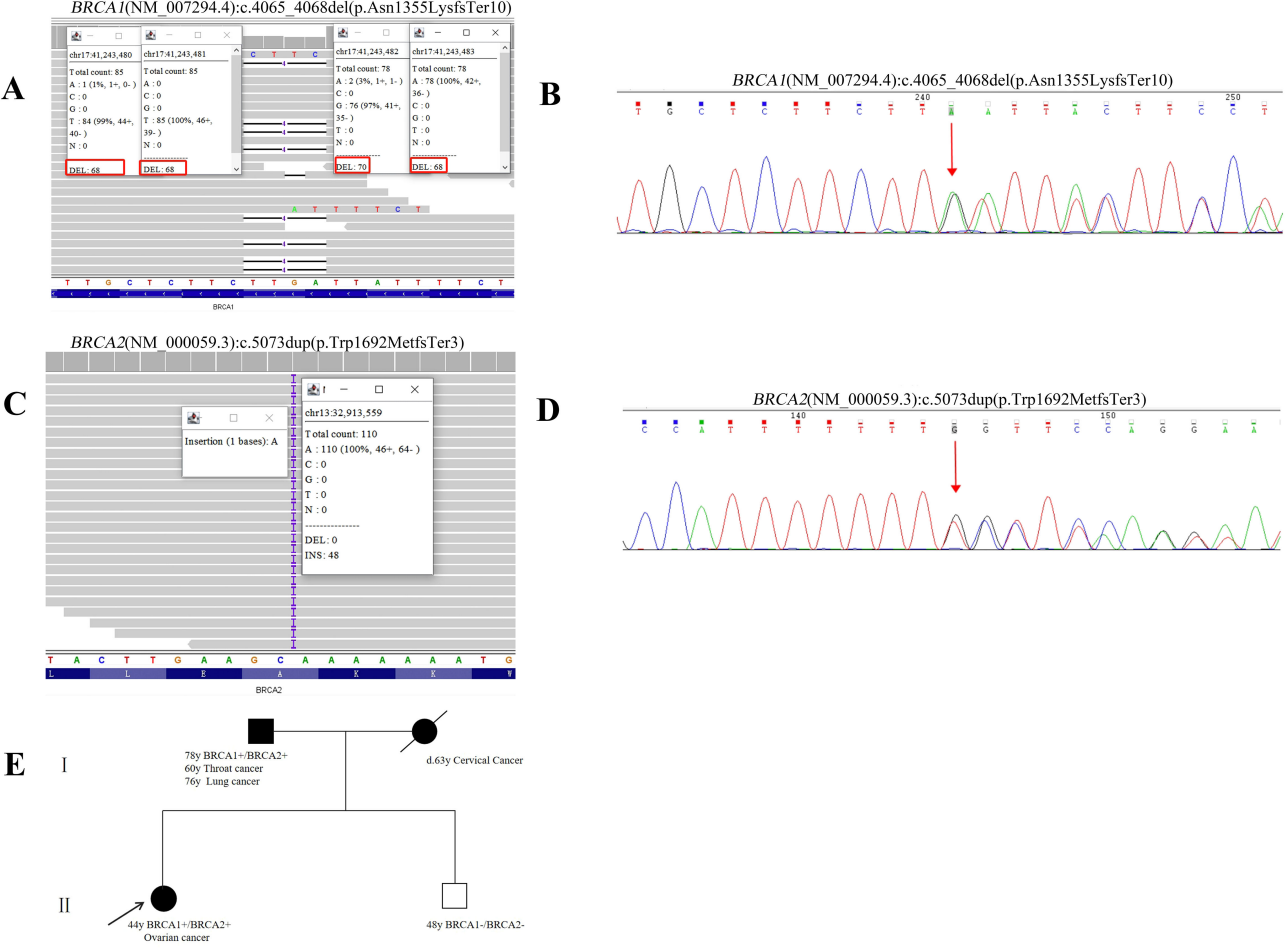
Pedigree and molecular test results of Patient 2. **(A)** WES data of the detected *BRCA1* (NM_007294.4):c.4065_4068del (p.Asn1355LysfsTer10) pathogenic variant. **(B)** Sanger sequencing result of the *BRCA1* pathogenic variant. **(C)** WES data of the detected *BRCA2* (NM_000059.3):c.5073dup (p.Trp1692MetfsTer3) pathogenic variant. **(D)** Sanger sequencing result of the *BRCA2* pathogenic variant. Red arrows indicate the identified variants. **(E)** Pedigree of the individual 2. Proband is indicated with an arrow. Black indicates individuals with cancer. Cancer type and age at diagnosis are reported. The age for genetic testing and age of death are also reported. Family members carrying the pathogenic/likely pathogenic variant are marked with *BRCA1*+, *BRCA2*+.

The female proband was diagnosed with high-grade serous ovarian cancer (HGSOC) at the age of 44. In February 2020, she presented with abdominal distension, dull lower abdominal pain, and constipation. The patient had no significant medical comorbidities, no history of long-term medication use, infectious diseases, or allergies. Imaging revealed bilateral ovarian masses, pelvic peritoneal thickening, and ascites consistent with malignant ovarian tumors and metastasis. The patient underwent open abdominal cytoreductive surgery for OC, including total hysterectomy, bilateral salpingo-oophorectomy, appendectomy, omentectomy, resection of peritoneal lesions in the pelvic and abdominal cavities, and pelvic and para-aortic lymphadenectomy. Small residual miliary lesions remained on the diaphragm and mesentery, achieving R1 resection. According to the operative and pathological findings, the final pathological FIGO stage was determined to be IIIC. The patient underwent six cycles of TP regimen chemotherapy (paclitaxel combined with cisplatin) following surgery. Genetic testing identified two pathogenic variants: *BRCA1* (NM_007294.4) c.4065_4068del (p.Asn1355LysfsTer10) and *BRCA2* (NM_000059.4) c.5073dup (p.Trp1692MetfsTer3) (**Figure 3**). Based on ACMG guidelines, both variants were classified as pathogenic. Targeted therapy with Olaparib was initiated in August 2020, with dosing adjusted for hematologic parameters. Three-dimensional models of the protein structures were generated to provide additional insight into the structural consequences of these variants, as depicted in **Figures 2C, D**. The patient completed her last cycle of chemotherapy in July 2020 and has been followed up for 53 months, with no evidence of disease recurrence as of December 2024.

The proband underwent genetic counseling, during which a family pedigree was constructed. Sanger sequencing confirmed that her father carried the same genetic variant, while her brother tested negative. The family history revealed the mother had cervical cancer at the age of 63, and the father was diagnosed with throat cancer at the age of 60 and lung cancer at the age of 76. Another brother remains unaffected. Details of the familial cancer history are depicted in **Figure 3E**.

### 2.3 Discussion

This study is the first to report *BRCA1/BRIP1* GDH in OC and evaluates its clinical impact and treatment response. Additionally, a *BRCA1/BRCA2* GDH case is included for comparative analysis. Marked differences in treatment response and toxicity profiles were observed between the two patients. The patient with *BRCA1/BRIP1* GDH demonstrated substantial chemotherapy-induced toxicity and postoperative complications, suggesting compromised treatment tolerance. In contrast, the *BRCA1/BRCA2* GDH patient exhibited better chemotherapy tolerance. Both patients experienced clinical benefit from Olaparib maintenance therapy, achieving disease stabilization for 17 months in the *BRCA1*/*BRIP1* GDH patient and 53 months in the *BRCA1/BRCA2* GDH patient, respectively. Continued follow-up may reveal differences in long-term therapeutic outcomes. These findings highlight the influence of different GDH combinations on chemotherapy tolerance and efficacy. However, due to the extremely small sample size, these observations should be interpreted as hypothesis-generating and require validation in larger patient cohorts to elucidate their underlying biological and clinical implications. In addition to genetic factors, differences in clinical presentation and prior treatments may have contributed to the distinct outcomes observed in our two patients. Patient 1 was diagnosed at a more advanced FIGO stage with evidence of bone metastases and had received prior bevacizumab therapy, which may have negatively impacted treatment tolerance and increased the risk of severe toxicity. Patient 2 was diagnosed at an earlier stage and did not have bone metastases or prior exposure to bevacizumab, potentially contributing to better treatment tolerance and efficacy.

Among OC patients receiving first-line platinum-based chemotherapy, germline *BRCA* gene variants have been associated with an increased incidence of grade 2/3 anemia (18). The present case, which presented with rare postoperative chylous ascites and progressed to grade 4 anemia. These observations raise the possibility that combined *BRCA1/BRIP1* gene deficiency could be associated with increased platinum-induced toxicity. The RING domain (amino acids 8–96) and BRCT domain (1646–1859) of *BRCA1* are essential for homologous recombination (HR) (19). *BRIP1*, through its BRCT domain (888–1063), interacts with *BRCA1* and plays a pivotal role in the Fanconi anemia (FA) and HR repair pathways (5). FA pathway is a crucial DNA repair pathway primarily responsible for recognizing and repairing DNA interstrand cross-link (ICL) damage, thereby maintaining genomic stability (20). The *BRCA1* (NM_007294.4) c.3288_3289del (p.Leu1098SerfsTer4) variant is predicted to disrupt the functionality of both its RING and BRCT domains, while the *BRIP1* (NM_032043.3) c.3072del (p.Ser1025HisfsTer34) variant may impair its interaction with *BRCA1*. This dual defect could significantly impair the ability to repair DNA damage induced by chemotherapeutic agents, such as platinum compounds. Furthermore, variation in *BRIP1* may affect hematopoietic function, thereby increasing the risk of hematological toxicity following treatment. Our findings provide new insight into the severe gastrointestinal and hematological toxicities observed in this patient. However, as this observation is derived from a single case and this study did not experimentally investigate the functional impact of these genetic alterations, it should be considered hypothesis-generating. Future research confirmation in larger, well-characterized patient cohorts is warranted

A narrative review of reported cases suggests that the average age of diagnosis for OC patients with GDH is 46.7 years (**Table 1**). Compared to patients with a single *BRCA1/2* germline variant, GDH carriers tend to develop OC at a younger age (35). This observation aligns with our finding of early onset in GDH patients. While GDH involving *BRCA1/2* has been associated with younger age at diagnosis and poorer prognosis in breast cancer patients (36), other studies have found no statistically significant differences in prognosis (37, 38). In our study, Patient 2 exhibited a stable disease course, supporting the latter perspective, whereas Patient 1 had a more aggressive disease trajectory with heightened chemotherapy-related toxicity. This suggests that different GDH combinations may differentially impact clinical outcomes. Notably, analysis of the cases summarized in **Table 1** reveals that most GDH-associated OC patients present with advanced-stage disease, predominantly FIGO stage III, and the majority of these cases are reported from European populations. Data from Asian cohorts remain limited. Furthermore, while prior literature has primarily focused on the genetic characterization of GDH, detailed descriptions of treatment strategies are relatively scarce. Nevertheless, available follow-up data suggest that the overall survival in these patients may be generally favorable. Given the pronounced chemotherapy-related toxicity observed in patients harboring *BRCA1/BRIP1* GDH, it is clinically prudent to consider early implementation of individualized supportive care strategies. These may include the prophylactic administration of hematopoietic growth factors and tailored nutritional support to mitigate hematologic and gastrointestinal adverse events. Furthermore, in cases of poor chemotherapy tolerance, reduced-intensity regimens or earlier initiation of targeted therapies, such as PARP inhibitors, may serve as feasible alternatives to enhance tolerability without compromising therapeutic efficacy. Although these considerations remain preliminary, they underscore the necessity of developing personalized treatment approaches informed by specific GDH genotypes. However, the rarity of GDH cases, particularly within Asian populations, poses a challenge in drawing generalized conclusions. Future studies with larger cohorts are necessary to systematically compare the effects of different GDH combinations and their implications for patient management.

**Table 1:** Previous and the present double heterozygosity variants identified in ovarian cancer.

No.	Geographic	Age	Clinical Profile and Follow-up Information				Variant 1		Variant 2		Year	Reference
	Area	Age at diagnosis of OC and BC	Histologic subtype	FIGO stage	Drug Therapy	Follow-up Duration(years or months)	Gene 1	DNA	Gene 2	DNA		
1	Hungarian	OC: 50 BC: 48	Ovarian adenocarcinoma; Infiltrating ductal breast carcinoma	unknown	unknown	unknown	*BRCA1*	c.185delAG	*BRCA2*	c.6174delT	1997	(21)
2	Ashkenazi Jewish	OC: 57 BC: unknown	unknown	OC: IV BC: unknown	unknown	OC: 5 years	*BRCA1*	c.185delAG	*BRCA2*	c.6174delT	1998	(22)
3	Ashkenazi Jewish	OC: 41 BC: 30	Papillary serous ovarian cystadenocarcinoma; Infiltrating ductal breast carcinoma	OC: IIIC BC: I	unknown	unknown	*BRCA1*	c.3888delGA	*BRCA2*	c.6174delT	1998	(23)
4	Netherlands	OC: 40 BC: 45	Papillary serous ovarian carcinoma; Infiltrative ductal breast carcinoma	OC: IIb BC: unknown	unknown	OC: 5 years BC: unknown	*BRCA1*	c.2804delAA	*BRCA2*	c.3715delG	2005	(24)
5	Ashkenazi Jewish	OC: 57 BC: 46	Serous ovarian carcinoma; Invasive ductal breast carcinoma	unknown	OC: paclitaxel and carboplatin; BC: 5-fluorouracil, doxorubicin, and cyclophosphamide	OC: 8 months BC: 11 years	*BRCA1*	c.5382insC	*BRCA2*	c.6174delT	2007	(25)
6	Italy	OC: 42 BC: 38	Papillary ovarian adenocarcinoma; Intraductal carcinoma	unknown	unknown	OC: 2 years BC: 6 years	*BRCA1*	c.5382insC	*BRCA2*	c.6024delTA	2010	(26)
7	Danish	OC: 59 BC: 53	unknown	unknown	unknown	OC: unknown BC: 6 years	*BRCA1*	c.5096G>A	*BRCA2*	c.631 + 4A > G	2010	(27)
8	Northern Italy	OC: 36 BC: 30	Papillary serous ovarian carcinoma; Ductal breast cancer, medullar type	OC: II BC: unknown	OC: carboplatin and taxol collaterally; BC: cyclophosphamide, methotrexate, and 5-fluorouracil	OC: 4 years BC: 10 years	*BRCA1*	c.4035delTT	*BRCA2*	c.5608delG	2010	(28)
9	Northern Italy	OC: 58 BC: 46	Papillary serous ovarian cystadenocarcinoma; Infiltrating ductal breast cancer	OC: IIIC BC: unknown	OC: carboplatin and paclitaxel BC: no	OC: 6 years BC: 18 years	*BRCA1*	c.1806C>T	*BRCA2*	c.6697C>T	2010	
10	Northern Italy	OC: 52 BC: 52	Serous ovarian adenocarcinoma; Ductal breast cancer	OC: IIIC BC: II	OC/BC: carboplatin and paclitaxel	OC/BC: 29 months	*BRCA1*	c.2524delTG	*BRCA2*	c.4512insT	2010	
11	German	OC: 50 BC: No	Papillary serous ovarian carcinoma	OC: IV	unknown	OC: 2 years	*BRCA1*	c.2080delA	*BRCA2*	c.1672delC	2011	(29)
12	Northern Italy	OC: 39 BC: 35	Ductal breast cancer	OC: unknown BC: III	unknown	OC: 7 years BC: 11 years	*BRCA1*	c.300T>G	*MLH1*	c.1480dupC	2013	(30)
13	China	OC: 56 BC: unknown	unknown	OC: IIC BC: unknown	unknown	unknown	*BRCA1*	c.1214C>G	*BRCA2*	c.3883C>T	2019	(31)
14	Italy	OC: 45 BC: No	High-grade serous ovarian cancer	unknown	unknown	unknown	*BRCA1*	c.3756_3759delGTCT	*APC*	c.3927_3931delAAAGA	2020	(32)
15	Northern Italy	OC: 36 BC: No	unknown	unknown	unknown	unknown	*BRCA1*	c.3752_3755delGTCT	*BRCA2*	c.425 + 2T>C	2020	(9)
16	American	OC: 53 BC: No	unknown	unknown	unknown	unknown	*MLH1*	c.1717_1718delGT	*BRCA2*	c.3942dupT	2021	(33)
17	Japan	OC: 28 BC: No	Mucinous adenocarcinoma of the ovary	unknown	unknown	OC: 9 years	*MSH2*	exon7dup	*BRCA2*	c.8016delA	2023	(34)
18	China	OC: 46 BC: No	Ovarian adenocarcinoma	OC: IVB	paclitaxel, cisplatin, and bevacizumab; paclitaxel and carboplatin; paclitaxel, carboplatin, and bevacizumab; Olaparib	17 months	*BRCA1*	c.3288_3289del	*BRIP1*	c.3072del	2024	Current report
19		OC: 41 BC: No	High-grade serous ovarian cancer	OC: IIIC	paclitaxel and cisplatin; Olaparib	53 months	*BRCA1*	c.4065_4068del	*BRCA2*	c.5073dup		

OC, ovarian cancer; BC, Breast cancer; unknown, Not mentioned in the literature.

Notably, individuals from two families carrying germline variants in *BRCA* and *BRIP1* exhibited a spectrum of malignancies, including esophageal cancer, renal cancer, ovarian cancer, laryngeal cancer, and lung cancer. While the association between OC and *BRCA/BRIP1* germline variants is well established, the relationship between these variants and other malignancies requires further investigation. Our findings provide additional insights that may inform future research in this area.

### 2.4 Conclusion

This study is the first to characterize *BRCA1/BRIP1* GDH variants in OC and compare their clinical impact with *BRCA1/BRCA2* GDH variants. Our findings highlight the differential impact of GDH combinations on chemotherapy tolerance and therapeutic outcomes. The results suggest that patients with *BRCA1/BRIP1* GDH variants may require personalized dose modifications for platinum-based chemotherapy to minimize toxicity while maximizing efficacy. Simultaneously, more frequent complete blood count monitoring and early or prophylactic administration of hematopoietic growth factors, along with nutritional support, may be necessary to mitigate hematologic toxicity. However, given the limited sample size (n=2) and the absence of functional validation, these results should be interpreted with caution and considered as hypothesis-generating. Further functional and clinical studies are necessary to further elucidate the underlying molecular mechanisms and refine treatment strategies for GDH-associated OC. Expanding the sample size and integrating functional analyses will be essential in optimizing individualized therapeutic approaches for patients with GDH variant.

## Data Availability

The datasets presented in this study can be found in online repositories. The names of the repository/repositories and accession number(s) can be found in the article/**Supplementary Material**.
