# Supplementary material for: Case Report: Clinical impact of BRCA1 and BRIP1 vs. BRCA1 and BRCA2 germline double heterozygosity in ovarian cancer: a comparative case study
Source: Front Oncol. 2025 Jul 24;15:1614373. doi: 10.3389/fonc.2025.1614373 (PMC12328145; doi:10.3389/fonc.2025.1614373)
Supplement: Supplementary file 1 [file Table1.docx]

Supplementary Material

Clinical Impact of BRCA1 and BRIP1 vs. BRCA1 and BRCA2 Germline Double Heterozygosity in Ovarian Cancer: A Comparative Case Study

# Supplementary Table

**Supplementary Table 1: Primers for *BRCA1* c.3288_3289del, *BRIP1* c.3072del, *BRCA2* c.5073dup and *BRCA1* c.4065_4068del.**

| No. | Gene and variant | chrom | start | end | ForwardPrimer（5'-3'） | ReversePrimer（5'-3'） |
| --- | --- | --- | --- | --- | --- | --- |
| 1 | *BRCA1* c.3288_3289del | chr17 | 41243923 | 41244484 | ATTTCTTGGCCCCTCTTCGG | ACAGTGAGCACAATTAGCCGT |
| 2 | *BRIP1* c.3072del | chr17 | 59761200 | 59761629 | TTGAGGGCATGATCCAAACGAT | TCGTTGGGGGCTCTAAGTTATG |
| 3 | *BRCA2* c.5073dup | chr13 | 32913238 | 32913736 | CATTGAGATCACAGCTGCCC | GCTGTTAGACATGCTACTGTTAC |
| 4 | *BRCA1* c.4065_4068del | chr17 | 41243213 | 41243627 | ACCAGAAGTAAGTCCACCAGT | TGCAAATACAAACACCCAGGA |
